# Black spicules from a new interstitial opheliid polychaete *Thoracophelia minuta* sp. nov. (Annelida: Opheliidae)

**DOI:** 10.1038/s41598-020-80702-6

**Published:** 2021-01-15

**Authors:** Naoto Jimi, Shinta Fujimoto, Mami Takehara, Satoshi Imura

**Affiliations:** 1 National Institute of Polar Research, 10-3 Midori-cho, Tachikawa, Tokyo 190-8518 Japan; 2 Research Center for Marine Biology, Graduate School of Life Sciences, Tohoku University, 9 Asamushi, Aomori, 039-3501 Japan; 3 The Graduate University for Advanced Studies, SOKENDAI, 10-3 Midori-cho, Tachikawa, Tokyo 190-8518 Japan

**Keywords:** Zoology, Evolution, Taxonomy

## Abstract

The phylum Annelida exhibits high morphological diversity coupled with its extensive ecological diversity, and the process of its evolution has been an attractive research subject for many researchers. Its representatives are also extensively studied in fields of ecology and developmental biology and important in many other biology related disciplines. The study of biomineralisation is one of them. Some annelid groups are well known to form calcified tubes but other forms of biomineralisation are also known. Herein, we report a new interstitial annelid species with black spicules, *Thoracophelia minuta* sp. nov., from Yoichi, Hokkaido, Japan. Spicules are minute calcium carbonate inclusions found across the body and in this new species, numerous black rod-like inclusions of calcium-rich composition are distributed in the coelomic cavity. The new species can be distinguished from other known species of the genus by these conspicuous spicules, shape of branchiae and body formula. Further, the new species’ body size is apparently smaller than its congeners. Based on our molecular phylogenetic analysis using 18S and 28S sequences, we discuss the evolutionary significance of the new species’ spicules and also the species' progenetic origin.

## Introduction

Annelida is one of the most ecologically and morphologically diverse group of animals known from both marine and terrestrial environments. Several groups are highly specialised with distinct ecological niches such as interstitial, parasitic, pelagic, or chemosynthetic zones^1^. Like many other animal phyla^2–6^, annelids are known to produce biominerals^2^. Groups such as Serpulidae, Sabellidae, and Cirratulidae, forming calcium encrusted tubes^7–9^, are commonly encountered in the marine environment. However, biominerals produced as a part of the animal body (chaetae, body shields, granule-shaped inclusions, and rod-shaped inclusions) are only sporadically reported from distantly related annelid groups^10,11^, implying their multiple origins.

The family Opheliidae is one of those groups that have biomineralisation-acquired representatives. Species of this family are active burrowers with an elongate and cylindrical body and they are commonly found in sandy or muddy substrates from the intertidal zone to deep-sea floor^1^. The biominerals were reported by Belova & Zhadan (2011) in several species of the genus *Ophelia* with rod-like inclusions that are produced by cells floating in the coelom. However, the mineral composition of these inclusions was not investigated^12^.

We found an interstitial opheliid species with rod-like inclusions from Yoichi, Hokkaido, Japan. For its body divided into three regions and absence of lateral eyes^13^, the species is designated to the genus *Thoracophelia* and we describe this species as *Thoracophelia minuta* sp. nov. In addition, we investigated the mineral composition of the rod-like inclusions. This is the first report of biomineralization in *Thoracophelia*. The small size of the new species compared to those of its congeners is also noteworthy. With molecular phylogenetic analysis, we investigated the evolutionary significance of these two topics.

## Materials and methods

**Figure 1:**
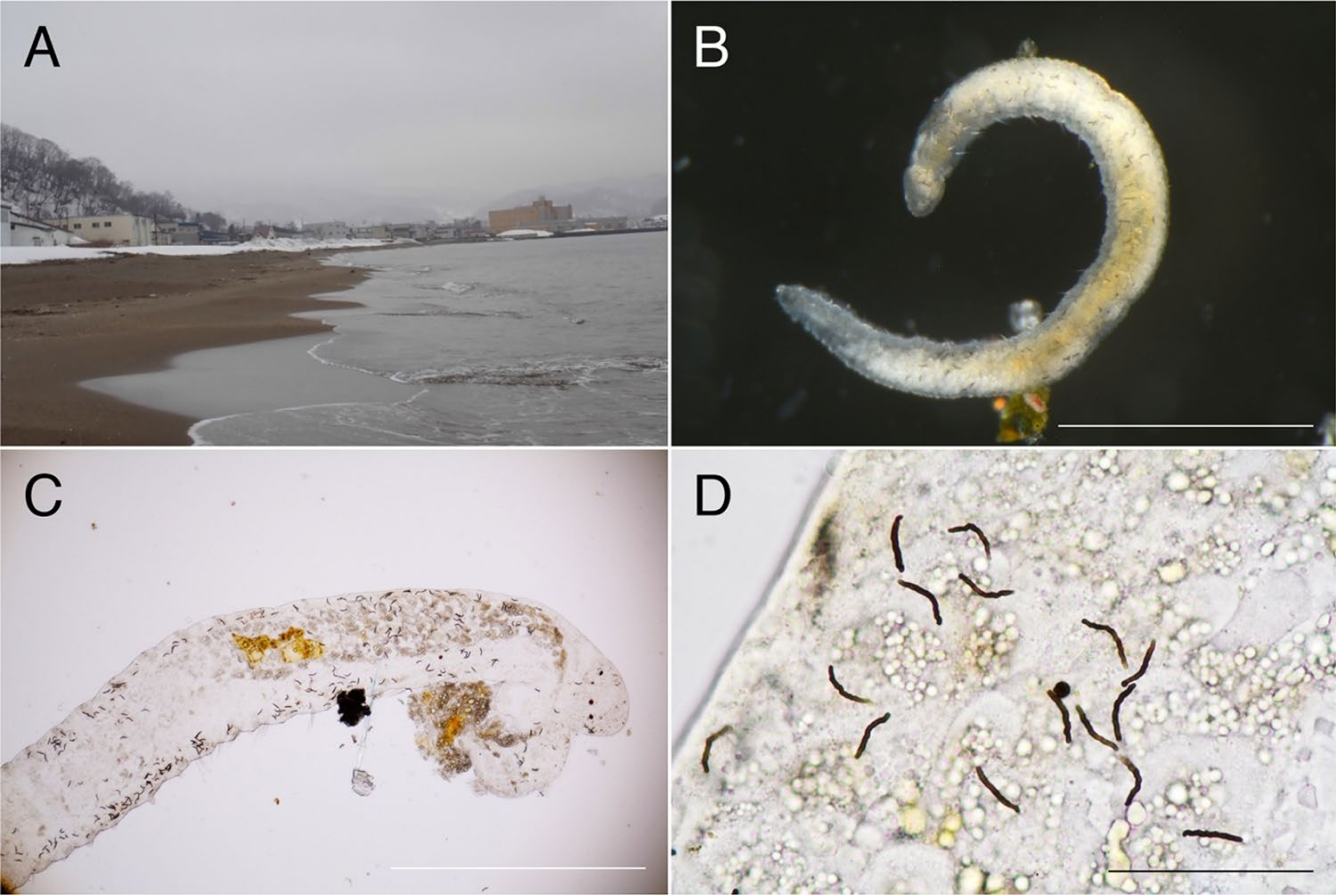
Habitat and live specimen of *Thoracophelia minuta* sp. nov. (**A**), the landscape of the type locality, Yoichi. (**B**), whole body, holotype. (**C**), Anterior end, paratype. (**D**), enlarged view of spicules, paratype. Scale bar: B, 0.5 mm; C, 250 μm; D, 100 μm. The figure was created with Adobe Illustrator v CS6 and Photoshop v CS6.

Four specimens of opheliid polychaetes were collected from subtidal sand at Yoichi, Hokkaido, Japan (43°11′ 59″ N, 140°46′ 56″ E, Fig. 1A) on 12th March 2019. The sediment sample was vigorously stirred in tap water, and the supernatant was poured through a 32 μm mesh plankton net. The extract on the net was immediately emptied in a dish with seawater and sorted under a stereomicroscope. Three specimens for morphological examination were fixed in 10% seawater buffered formalin and preserved in 70% ethanol, and one specimen for DNA extraction was fixed in 99.5% ethanol. The three former specimens were observed under stereomicroscopes MZ16, (LEICA, Germany) and E600 (Nikon, Japan). Two of the specimens were used for scanning electron microscope (SEM) observations. They were washed in deionised water, dehydrated in a graded ethanol series, dried in a critical-point dryer (HITACHI HCP-2) using liquid CO_2_, and coated with gold in an ion sputter (HITACHI E-1045). Observations were conducted using an SEM instrument (HITACHI S-3000N).

The observation of major elements was carried out using a scanning electron microscope (SEM; JEOL JSM-5900LV), installed with an energy dispersive spectrometer (EDS; X-Max 20 mm^2^ silicon drift detector processed with AztecOne), at the National Institute of Polar Research, Japan. The samples were placed on an aluminium disc and coated with carbon. The electron beam current was approximately 2 nA at an acceleration voltage of 15 kV. The specimens were deposited at the National Museum of Nature and Science, Tsukuba (NSMT) and Invertebrate Collection of the Hokkaido University Museum (ICHUM). The terminology of Opheliidae follows that of Santos et al.^14^.

Genomic DNA was extracted from one specimen (total body) following the methods of Jimi and Fujiwara^15^. The cytochrome *c* oxidase subunit I (COI), 18S rRNA (18S), and 28S rRNA (28S) genes were amplified and sequenced using the following primer sets: polyLCO (5′-GAYTATWTTCAACAAATCATAAAGATATTGG-3′) and polyHCO (5′-TAMACTTCWGGGTGACCAAARAATCA-3′)^16^; mitchA (CAACCTGGTTGATCCTGCCAGT) and mitchB (TGATCCTTCCGCAGGTTCACCTAC)^17^; and LsudiF (ACCCGCTGAATTTAAGCATA) and D3aR (ACGAACGATTTGCACGTCAG)^18^. The newly obtained sequences were deposited at the DDBJ/EMBL/GenBank (MW429791, COI gene, 677 base pairs (bp); MW429485, 18S gene, 1629 bp; MW429484, 28S gene, 1010 bp) (Table 1). The comparative sequences were retrieved from Paul et al. (2010)^19^ (Table 1). All sequences of 18S and 28S were aligned using MAFFT ver. 7.205 under the E-INS-i strategy^20^. Alignment-ambiguous positions were removed using trimAL under the gappyout strategy^21^. The trimmed sequences of the two genes, 18S (1019 bp) and 28S (713 bp), were concatenated using Kakusan^22^, which recommended a GTR + G evolutionary model for each of the genes. A phylogenetic tree was constructed using the maximum likelihood (ML) method in the RAxML-VI-HPC^23^ program. The robustness of the ML tree was evaluated by 1,000 bootstrap pseudo-replicates (-f option). Bayesian inference (BI) analysis was conducted using Mr. Bayes ver. 3.2.2^24^, with Markov chains of 10 million generations. The model choice for each partition was also based on the Kakusan results. Run convergence was analysed using Tracer ver. 1.6^25^; the first 1 million generations of trees were discarded as burn-in.

**Table 1:** List of opheliids and outgroup species included in the phylogenetic analysis, together with accession numbers in GenBank.

Species	18S	28S
*Armandia bilobata*	DQ779641	DQ779676
*Armandia brevis*	KF511818	KF511838
*Armandia maculata*	AY040681	HM746737
*Lobochesis bibranchia*	AB106266	–
*Ophelia bicornis*	AF508122	HM746745
*Ophelia neglecta*	AF448156	HM746747
*Ophelia rathkei*	AF448157	AY366513
*Ophelina acuminata*	HM746735	HM746744
*Ophelina cylindricaudata*	KF511824	KF511848
*Polyophthalmus pictus*	AF448161	AF185194
*Thoracophelia dillonensis*	KF511830	KF511851
*Thoracophelia ezoensis*	HM746725	HM746738
*Thoracophelia minuta*	MW429485	MW429484
*Thoracophelia mucronata*	KF511831	KF511852
*Thoracophelia williamsi*	KF511832	KF511854
*Neolipobranchius* sp.	AY612616	AY612626
*Sclerobregma branchiata*	AY612615	AY612623

For nomenclatural acts, we registered this publication in Zoobank. Zoobank LSID is urn:lsid:zoobank.org:pub:B38565DE-1445-402C-9CC2-4ED5161197C7.

## Results

### Observation of biominerals

**Figure 2:**
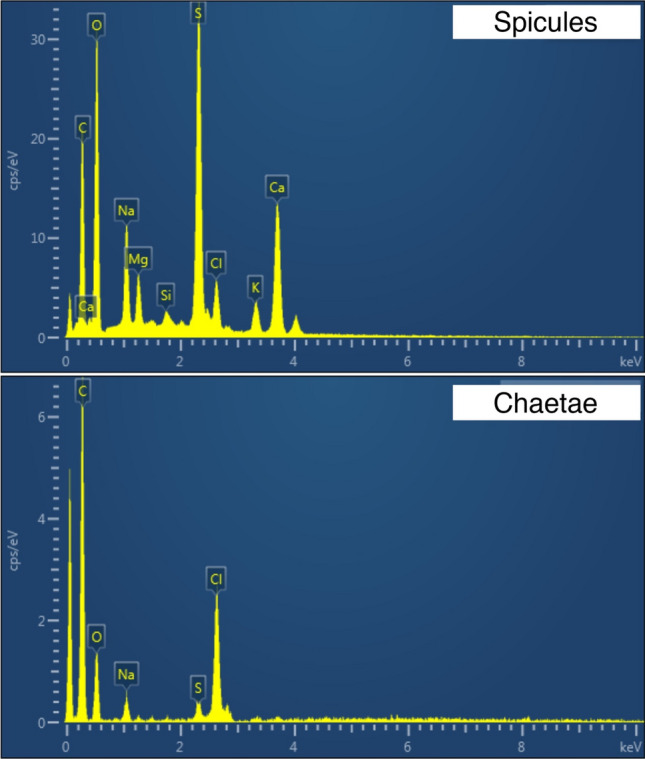
Results of energy-dispersive spectroscopy carried out on two tissue types in *Thoracophelia minuta* sp. nov. The figure was created with AZtecOne and Adobe Illustrator v CS6.

In the new species, approximately 300 rod-like inclusions were observed in the coelomic cavity filled with body fluid (Fig. 1B–D). They were neither attached to a specific organ nor embedded in tissue and drifted with the body fluid. Our observation of the raw material (without any staining) at 1000 × magnification using light microscopy could not determine whether the rod-like inclusions were intracellular or extracellular structures (Fig. 1C). This point was not clarified by the SEM as well. Although we were able to partly expose the rod-like inclusions by dissecting the body, we could not isolate them or distinguish between associated cells and artefactual presence of tissue. The rod-like inclusions had the following features (Fig. 1D): *i*) black colour, *ii*) approximately 20 μm in length, and *iii*) corrugated rod shape with some of them curved. SEM–EDS analysis detected high levels of carbon, oxygen, sulphur, and calcium from spicules, in contrast to chaetae with a high content of carbon, oxygen, and chloride (Fig. 2). However, carbon was sputtered during specimen preparation, and the detected carbon peaks were not reliable.

### Molecular phylogenetic analysis

**Figure 3:**
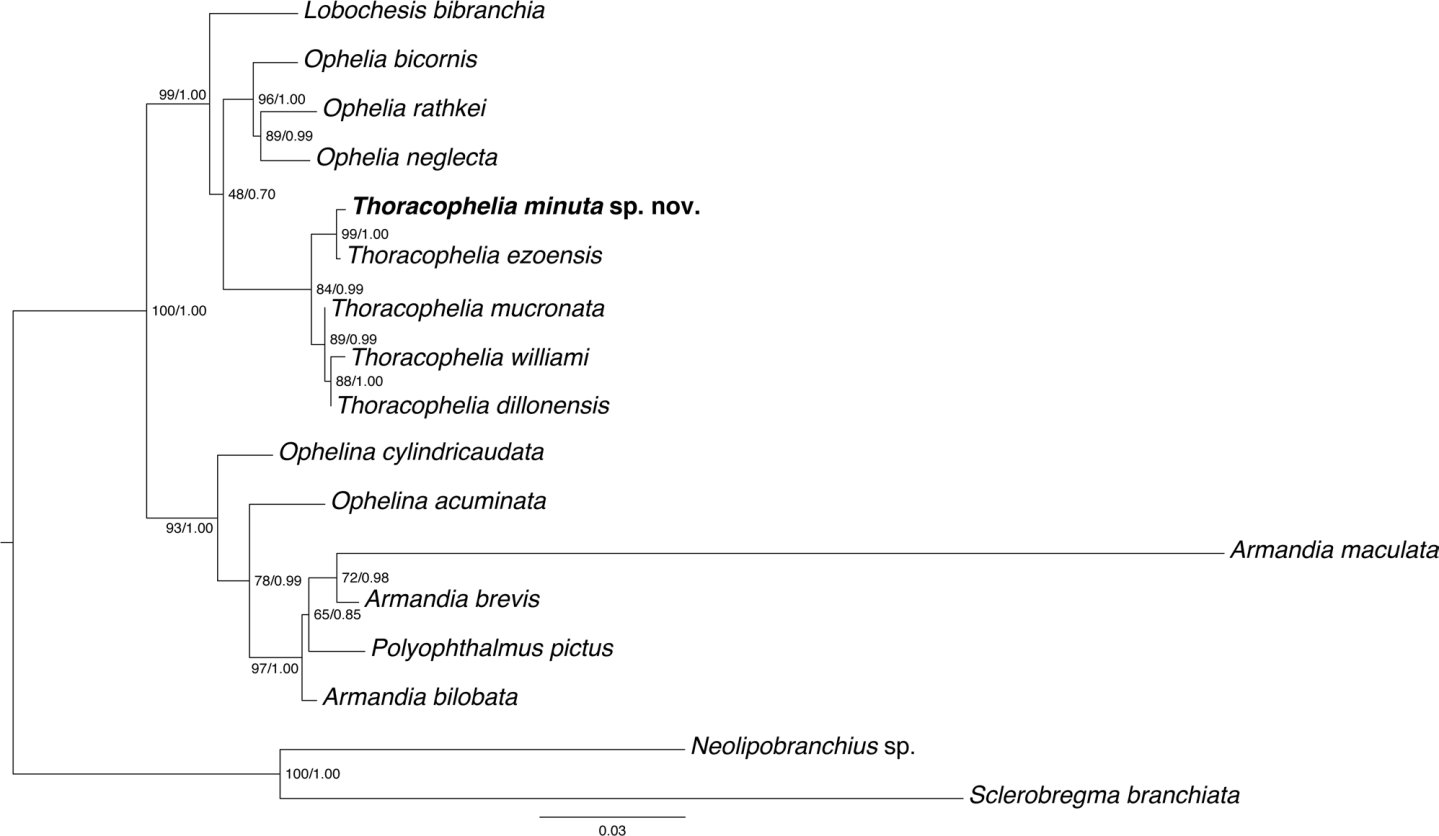
Maximum-likelihood (ML) phylogenetic tree of Opheliidae based on 18S and 28S sequences. *Neolipobranchius* sp. and *Sclerobregma branchiata* were used as ‘outgroups’ for the rest of the opheliids. Nodal support values (bootstrap support [BS] value) higher than 50% are indicated on each branch. The figure was created with Adobe Illustrator v CS6.

ML and BI analyses recovered the same topology. The monophyly of Opheliidae was inferred with maximum support values (100% bootstrap support [BS], 1.00 posterior probability [PP]), and the two main clades, the Ophelininae clade and the Opheliinae clade, were also inferred with high support values (93% BS, 1.00 PP; 99% BS, 1.00 PP), as in previous studies (Paul et al., 2010). The new species collected in this study, *Thoracophelia minuta* sp. nov., was a sister species to *Thoracophelia ezoensis* with high support values (99% BS, 1.00 PP) within Opheliidae (Fig. 3).

#### Taxonomic account

Family Opheliidae Malmgren, 1867.

Genus *Thoracophelia* Ehlers, 1897.

*Thoracophelia minuta* sp. nov.

[New Japanese name: sunatsubu-ophelia].

#### Material examined

Holotype (NSMT-Pol H-837) and paratype (ICHUM-6177) are deposited in the National Museum of Nature and Science, Tsukuba (NSMT). Other specimens were used for DNA extraction and spicule extraction.

#### Sequences

Sequences were determined from a specimen collected with the type material: COI, 677 bp, MW429791; 18S, 1629 bp, MW429485; 28S, 1010 bp, MW429484.

#### Description of holotype (variation amongst paratypes indicated in parentheses)

**Figure 4:**
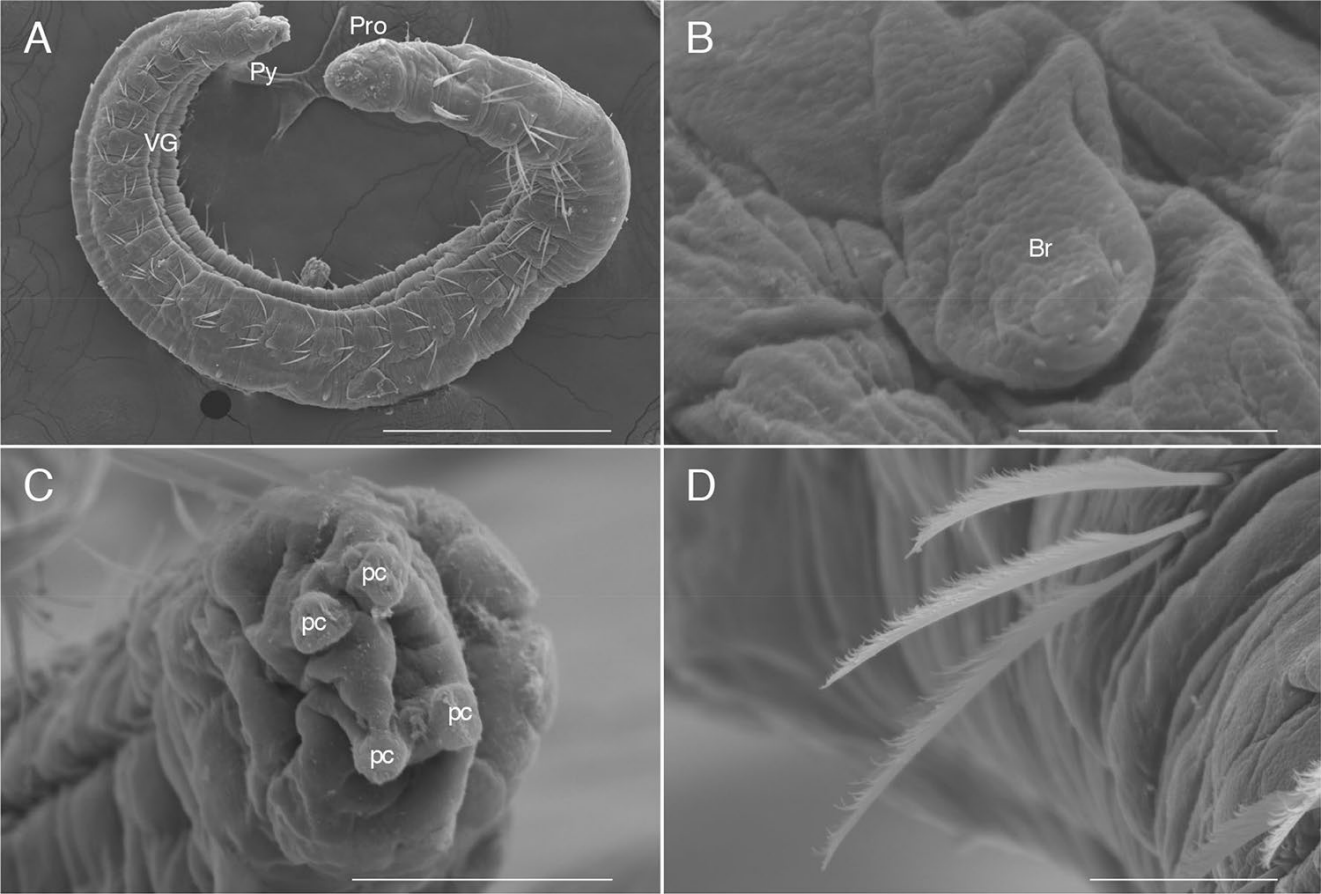
SEM photographs of *Thoracophelia minuta* sp. nov., paratype. A, whole body. B, branchia. C, pygidium. D, chaetae. Scale bars: A, 300 μm; B, 10 μm; C, 50 μm; D, 20 μm. Abbreviations: Br, branchia; pc, pygidial cirri; Pro, prostomium; Py, pygidium; VG, ventral groove. The figure was created with Adobe Illustrator v CS6 and Photoshop v CS6.

Preserved material white; color in life transparent. Holotype with gametes. Body 1.6 mm long (1.4 mm, n = 2), 0.1 mm width (0.1 mm, n = 2), 25 chaetigers (25, n = 2). Segmentation not clearly defined. Prostomium pointed, with 4 red eyes (4, n = 2) (Fig. 1C). Peristomium achaetous, cephalic region without chaetigers before cephalo-thoracic constriction (Figs. 1B, 4A). Chaetiger 1 biramous (Fig. 4A). Body formula 5a (abranchiate) + 20b (branchiate) + 0a (posterior abranchiate) (Fig. 4A). Pygidial funnel short, with 4 cirri of equal size on each side (Fig. 4C). Ventral groove extending from chaetiger 3 to pygidium. No lateral cirri or ridge in chaetiger 10. Noto- and neurochaetae all serrated chaetae (Fig. 4D), 2–3 chaetae per parapodium in chaetigers 1–24, one chaeta per parapodium in chaetiger 25. Noto- and neurochaetae in anterior segments longer than posterior ones. Branchiae simple, hemisphere shape (Fig. 4B). Segmental eyes absent. Oocytes present in the body (Fig. 1B), 10 μm in diameter. Body contained many spicules in coelom cavity (Fig. 1B,C,D).

#### Etymology

The new specific name derives from its minute body.

#### Habitat and distribution

Specimens of *Thoracophelia minuta* sp. nov. were collected from the subtidal beach of Yoichi, Hokkaido Prefecture, Japan. The species is only known from the type locality.

#### Remarks

This species is distinguished from other congeners by the following features: *i*) having a minute body (body length approximately 1. 5 mm), *ii*) simple hemisphere-shaped branchiae, *iii*) body formula 5a + 20b + 0a. Known species of *Thoracophelia* (See Table 1 of Santos et al. 2004^14^) have *i*) larger bodies (9–100 mm), *ii*) branched branchiae, *iii*) body formula 10–13a + 14–22b + 5–11a. The new species has minute body unlike other opheliids. However, since it has eggs, we can judge that it is a small but mature individual.

In Japan, four species of *Thoracophelia* have been recorded^26^: *Thoracophelia arcticus*, *T*. *ezoensis*, *T*. *japonicus*, *T*. *yasudai*. These species have a lateral glandular ridge on chaetiger 10 and branched branchiae. The new species do not have them. For understanding the biodiversity of the Japanese coast, investigation of interstitial species is also needed.

## Discussion

Among biominerals produced by animals, spicules are one of the common forms known in phyla such as Porifera, Cnidaria, Annelida, Mollusca, Nemertea, Platyhelminthes, Arthropoda, Echinodermata, and Urochordata^27^. Kingsley (1984) defined "spicules" as individual minute inclusions of calcium carbonate which occur over an extended region of the animal's body. Based on the result of the EDS analysis revealing a calcium-rich composition for the rod-like inclusions of *Thoracophelia minuta* sp. nov., we identify the new species’ rod-like inclusions as spicules. It is also important to note that the high S peak may be due to the simultaneous emission of sulphur during biomineralization since this species lives in the sand interstices, which suggests that it is susceptible to a reductive condition.

In annelids, various biominerals have been reported (Table 2). However, only three polychaete species (*Echinofabricia alata*, *E*. *dubia*, and *E*. *goodhartzorum* from the family Fabriciidae) have been known to possess spicules^28^. In opheliids, rod-like inclusions have been known in a previous study by Belova and Zhadan^12^. These inclusions are similar to the spicules of the new species in their shape and position in the body, except that the species observed by Belova and Zhadan^12^ have the inclusions growing out of cells. Further, Belova and Zhadan^12^ did not investigate the inclusions’ composition, preventing its identification as spicules.

**Table 2:** List of biomineralized materials in annelids.

Groups	Type of materials	Position	Main minerals	References
Amphinomidae	Chaetae	External	Ca	Pleijel et al.^11^
Sternaspidae	Ventral shields	External	Fe, P	Lowenstam^10^
Siboglinidae	Chaetae	external	Fe, Mn	Duperron et al.^29^
Cirratulidae	Tubes	External	Ca	Taylor et al.^7^
Sabellidae	Tubes	External	Ca	Vovelle et al.^8^
Serpulidae	Tubes	External	Ca	Vinn et al.^9^
Syllidae	Granule-shaped inclusions	Internal	Ca	Briggs et al.^30^
Nephtyidae	Granule-shaped inclusions	Internal	Ca	Gibbs and Bryan^31^
Fabriciidae (*Echinofabricia* spp.)	Rod-shaped inclusions	Internal	Ca	Huang et al.^28^
Opheliidae (*Ophelia* spp.)	Rod-shaped inclusions	Internal	?	Belova & Zhadan^12^
Opheliidae (*Thoracophelia minuta*)	Rod-shaped inclusions	Internal	Ca, S	This study

The molecular phylogenetic tree generated in this study confirmed that our spicule-possessing species, *Thoracophelia minuta* sp. nov., is nested in Opheliidae (Fig. 4). Opheliidae and Fabriciidae, the other family with spicule-possessing species, have never been grouped together in a clade^1^. Recent phylogenetic studies based on expressed sequence tag (EST) libraries also indicated that these two families do not form a clade^32,33^. These results indicate that spicules were acquired at least twice in Sedentaria, Annelida (Table 2). In the present study, the SEM–EDS identified the spicules' elemental composition and molecular phylogenetic analysis revealed the phylogenetic position of the new spicule-bearing species. However, to unravel the evolution of biomineralisation in Annelida, comparative histological studies of the biomineral-producing cells using a transmission electron microscope and further investigation for taxa with biominerals are necessary.

In addition to the distribution of biominerals in annelids, miniaturisation that took place in the new species might be noteworthy to understand the evolutionary significance of acquiring spicules. The new species have a minute body, unlike all other known species of *Thoracophelia*. In the molecular tree, the new species forms a clade with *T*. *ezoensis*, a species described from the same area (Hokkaido, Japan) as the new species^34^. The other *Thoracophelia* clade consists of *T*. *mucronata*, *T*. *williami*, and *T*. *dillonensis*, all collected from the same area of Dillon Beach, USA. It is likely that the new species speciated around the Sea of Japan from the ancestral species of the *T*. *minuta*–*T*. *ezoensis* clade. The most conservative view is that the ancestral species is a large species like all known species of *Thoracophelia,* except for the new species, and miniaturisation occurred in this species. In annelid taxa, two hypotheses of miniaturising evolution have been reported: progenesis or stepwise miniaturisation^35^. For *T. minuta* sp. nov., progenesis is the more plausible of these two hypotheses. Progenesis theory holds that larval or juvenile stages of a larger ancestor has temporarily ceased somatic cell development and become sexually mature.^36^ Unlike the larger congeners, the new species have simple branchiae and four pygidium papillae, which are characters only known from a young worm of *T*. *mucronata*^37^. These morphological similarities with the juvenile support progenetic evolution of *T*. *minuta* sp. nov. Considering that some interstitial taxa acquired spicules (e.g., Bertiliellidae in Platyhelminthes, Rhodopemorpha in Molluscs)^38,39^, this progenetic evolution may have driven the acquisition of spicules in the new species as well. The most closely related taxa *T*. *ezoensis* inhabits similar environments and areas as this new species^34^, and how this speciation and progenetic evolution occurred, is yet to be studied.

## Conclusions

In this study, we described *Thoracophelia minuta* sp. nov. that has black-rod shaped spicules. We found that the spicules contain high Ca and are distributed in the coelomic cavity. The new species has a minute body unlike other opheliid congeners. Our phylogenetic analysis indicated that progenetic evolution occurred in the new species. The relationship between this progenetic evolution (≈ interstitial life) and spicule evolution is still unclear. Further case studies are needed to understand the evolution of spicules in annelids.
